# Management of bacterial blight of carrots by phenolic compounds treatment

**DOI:** 10.1371/journal.pone.0299105

**Published:** 2024-04-01

**Authors:** Eliška Hakalová, Dorota A. Tekielska, Jan Wohlmuth, Jana Čechová

**Affiliations:** Mendeleum–Department of Genetics, Faculty of Horticulture, Mendel University in Brno, Lednice, Czech Republic; Tocklai Tea Research Institute, INDIA

## Abstract

Bacterial blight is a serious disease of carrot production worldwide. Under favorable conditions, the causal organism *Xanthomonas hortorum* pv. *carotae* causes serious loss especially in seed production because of its seed-borne character. Unlike fungal diseases, the treatment of bacterial diseases is limited and methods such as hot water or sodium hypochlorite (bleach) treatment are mainly used by seed companies. Here, we compared the efficacy of hot water treatment, sodium hypochlorite treatment and treatment with three phenolic compounds–carvacrol, thymol and eugenol, to eliminate *Xanthomonas* growth *in vitro* and subsequently *in vivo* on seeds of Xhc low, medium and highly infested carrot seed lots. The complete elimination of Xhc from germinated plants was obtained only for Xhc low infested seed lot with 1% sodium hypochlorite and carvacrol solutions in concentrations of 0.0196%– 0.313%. The significant reduction of Xhc presence in germinated plants of Xhc medium infested seed lot was achieved with 1% sodium hypochlorite treatment and hot water treatment. However, hot water treatment resulted in a significant reduction of seed germination percentage as well. Considering the elimination of Xhc infection from germinated plants and the effect on seed germination and plant vigor, 0.0196% carvacrol solution was suggested as an alternative to 1% sodium hypochlorite treatment regarding additional costs related to the liquidation of used treated water and to hot water treatment that has been proved to be insufficient to obtain disease-free plants.

## Introduction

Starting cultivation of crops from pathogen-free, healthy seeds or transplants seems logical and obvious, yet it is unbelievably difficult to achieve. Seed borne bacterial pathogens are of particular concern because strategies for the management of bacterial diseases, unlike fungal diseases, are limited and not many effective treatments are available. Although seed production has been experiencing a boom in possibilities of eliminating seed pathogens, seed-borne bacterial diseases continue to be a serious problem and cause significant economic losses worldwide [1].

For carrot production, the pathogenic bacterium *Xanthomonas hortorum* pv. *carotae* (Xhc) is a common contaminant of seed lots [2]. Infested seeds serve as a primary inoculum of bacterial blight. Even when disease symptoms are not observed in carrot seed plants, seeds may become infested with Xhc from asymptomatic, epiphytic populations of the pathogen that resides on leaves and umbels. The manifestation of bacterial blight symptoms was described to happen when populations bigger than 10^6^ CFU per gram of leaf tissue develop on foliage or when the seed contamination exceeds 10^4^ to 10^5^ CFU per gram of seed [3, 4]. Xhc might be disseminated by rain, wind, or mechanically transferred during carrot cultivation. Moreover, the dissemination of bacterial inoculum through aerosolized crop debris was described [5]. Symptoms of bacterial blight consist of small, yellow, angular water-soaked lesions on leaves at the beginning of the infection that convert to dry, irregular brown spots with yellow surroundings. Dark-brown streaks on the petioles, peduncles and stems and blight on flowers were described as well [4, 6]. Xhc was considered an A1 plant pest by the Caribbean Plant Protection Commission and certification programs proposed requirements for production of disease-free plants. Currently, Xhc is marked as a quarantine pest in Mexico and Israel [7, 8]. Although yield losses may occur in carrot fields with high incidences of bacterial blight, the economic impact of the disease is considered to be minimal in general. However, the highest seriousness of this pathogen is presented in regard to the seed quality [9]. Regarding the host resistance, a germplasm screening of carrot species indicated that four PI lines and two of the wild relatives are highly resistant against Xhc [10]. Nevertheless, for current varieties, the elimination of the pathogen from seeds represents the main part of disease management.

The first step to clean, healthy seeds is the detection of bacterial infection in a seed lot. For this purpose, various seed health assays are used. In the case of Xhc, the International Seed Testing Association (ISTA) recommends a seed wash dilution-plating assay including washing seeds in buffer and plating serial dilutions of the extract on a semi-selective medium. Either a pathogenicity test or a polymerase chain reaction (PCR) is used to confirm suspect isolates [9, 11]. In 2019, the gel-based PCR assay [12] was replaced by a TaqMan assay consisting of two pairs of Xhc specific primers and probes targeting specific markers Xhc3S and Xhc02 [13, 14] and one set of universal primers and probe detecting a part of the *16S rRNA* gene [15]. However, presented Xhc specific assays were evaluated only for bacterial cultures and the suitability for its use in the case of carrot tissues was not described.

Effective microbial control practices have to reduce the microbial population without harming seed germination and growth and quality of germinated plants. For the suppression of seed-borne xanthomonads, the application of physical methods such as hot water treatment and dry heat or use of chemical compounds such as sodium hypochlorite or copper-based compounds are the prevalent ones [14, 16–18]. Based on the demands of ecological production, an increase in the application of biological compounds with antimicrobial activity was recently noticeable. From these, phenolic compounds like carvacrol, eugenol and thymol are reported as effective in suppressing *Xanthomonas* growth *in vitro* [19–22]. However, *in vivo* results are not often presented. The antimicrobial properties of carvacrol are related to its ability to destabilize a membrane structure followed by the increase of fluidity and permeability and release of ions, cellular material, ATP and nucleic acid from the cytoplasm [23, 24]. Similarly to carvacrol, thymol activity is connected with the interaction with membrane, membrane proteins and intracellular targets and leakage of potassium ions and ATP. Eugenol shows the ability to increase membrane nonspecific permeability, inhibit the activity of enzymes like ATPase, amylase or protease and interact with proteins [23–27].

The study presents the importance of detecting *Xanthomonas hortorum* pv. *carotae* in germinated plants and highlights the potential of the carvacrol compound for managing bacterial blight disease. The practical application of phenolic compounds as an alternative to hot water and sodium hypochlorite treatments is assessed through real-time PCR estimation of Xhc presence in plants germinated from naturally infested seeds, along with the observation of seed germination percentage and the vigor of the germinated plants. Simultaneously, the results point out the critical steps in the conversion of *in vitro* results into *in vivo* experiments and further into practical use.

## Materials and methods

### Pathogen detection and confirmation of *Xanthomonas hortorum* pv. *carotae* transmission to germinated plants

The detection of Xhc in carrot tissues was performed by a real-time PCR targeting the *hpaP* (hypersensitive response and pathogenicity associated phosphatase) gene using primers T3S_fwd (5’-CAATTGCCCTCATCTACGCA-3’) and hpaP_rev2 (5’-CTTCATGCAACTGCGACGAC-3’) [28]. A standard curve was achieved from a serial dilution of a DNA extract obtained from 1 mL of Xhc NCPPB 4410 suspension with the concentration of 10^8^ CFU mL^−1^ (based on OD_600_ and colony counting method). For dilutions, DNA from healthy carrot leaf tissues was used. As a SYBR Green approach was used, the confirmation of the melting temperature of a target product was performed on six isolates of Xhc used in this study. All reactions were carried out in triplicates using the Luna qPCR Master Mix (NEB, Ipswich, MA, United States) according to manufacturer’s instructions and the following thermal profile: 95°C for 2 min and 40 cycles of 95°C for 15 s, 60°C for 45 s and 70°C for 10 s. The amplification was immediately followed by melting analysis ramping from 80°C to 95°C in 0.5°C increments. The qTOWER3 instrument (Analytic Jena, Jena, Germany) and qPCRsoft v. 4.0 (Analytic Jena, Jena, Germany) were used.

The seeds of a carrot seed lot (*Daucus sativa* L., cv. Galaxy) positively detected for a presence of *Xanthomonas* infection by the endpoint PCR [12] were sown on sterile filter papers in the amount of 200 seeds in ten repetitions to confirm the transmission of the infection to the germinated plants. Two weeks after sowing, germinated plants without testas were homogenized in Bioreba bags (Bioreba, Reinach, Switzerland) with 2 mL of phosphate-buffered saline (PBS). Total genomic DNA of tested plants was extracted from 0.5 mL of a plant homogenate with the NucleoSpin Tissue kit (Macherey-Nagel, Düren, Germany) according to the manufacturer’s instructions. The detection of Xhc infection was performed by real-time PCR as described above.

### Bacterial strains and growth conditions

Bacterial cultures (listed in S1 Table) were obtained from the National Collection of Plant Pathogenic Bacteria (NCPPB, London, UK) and the Collection of Microorganisms of Mendeleum–Institute of Genetics (MEND, Lednice, Czech Republic). Except for six strains of *Xanthomonas hortorum* pv. *carotae*, two strains of *X*. *arboricola* isolated from carrot leaves and with confirmed pathogenicity on carrot cv. Napoli were also used. The isolates were grown in the dark on Luria Agar (LA, HiMedia, Mumbai, India) at 28°C overnight and bacterial suspensions of approximately 10^8^ CFU mL^-1^ (based on OD_600_) concentrations were prepared in Luria-Bertani broth (LB, HiMedia, Mumbai, India). For the antimicrobial analysis, the concentration of 10^6^ CFU mL^-1^ was used.

### Phenolic compounds

Carvacrol, eugenol and thymol were purchased from P-LAB (Prague, Czech Republic). Carvacrol and eugenol compounds were prepared as 10% stock solutions in LB broth supplemented by 1% dimethyl sulfoxide (DMSO, Penta, Chrudim, Czech Republic) to increase their solubility. Subsequently, compounds were serially diluted to the appropriate concentration in the same way. Thymol was first dissolved in 96% ethanol (maximum final presence of ethanol was 5% *v/v*) and subsequently diluted to the 10% concentration with pure LB broth. Serial dilutions were performed by pure LB broth as well.

### Antibacterial activity of phenolic compounds

The antibacterial activity of phenolic compounds was evaluated through the minimum inhibitory concentration (MIC) and the minimum bactericidal concentration (MBC) according to da Silva et al. [21] and by the time-kill assay [29], with modifications according to Hakalová et al. [19]. The minimum inhibitory concentration was defined as the lowest concentration of an antibacterial agent that completely inhibits the visible growth of a microorganism after overnight incubation. The minimum bactericidal concentration is presented as the lowest concentration of an antibacterial agent that prevents the growth of a microorganism after subculture to the antibiotic-free medium [30].

Briefly, MIC values were determined by the broth microdilution method in 96-well plates. Approximately 10^6^ CFU mL^-1^ of each *Xanthomonas* strain was inoculated into LB broth containing two-fold dilutions of the individual phenolic compounds (concentration range from 0.3125% to 0.0049%) in a 1:1 ratio. Phenolic compounds diluted by LB broth with 1% DMSO or non-supplemented LB broth were used as measurement controls, the concentration of 10^6^ CFU mL^-1^ of bacterial suspension diluted by pure LB broth served as the positive control. After 24h of treatment, the optical density was determined at 600 nm (OD_600_) using a microplate reader (SPECTROstar Nano, BMG Labtech, Ortenberg, Germany). The MBC values were determined by subculturing 5 μL from the broth dilution tests on LA without the antimicrobial agent, followed by the 24h cultivation at 28°C in the dark. All assays were carried out in triplicates.

The time-kill assay was carried out using the viable cell count method in three repetitions. Xhc strains NCPPB 425 and NCPPB 4410 representing strains with both, higher and lower susceptibility to the phenolic treatment evaluated within the first assay were used. Five mL of carvacrol and thymol solution in the concentration gaining the bactericidal effect on individual strains were combined with the corresponding bacterial suspension (approximately 10^6^ CFU mL^-1^ based on OD_600_) in a 1:1 ratio. Bacterial cultures diluted by LB supplemented by DMSO served as growth controls. At six time points (0 s, 30 s, 10 min, 20 min, 30 min and 40 min) of incubation, 100 μL of cultures were serially diluted and cultivated on LA in triplicates. The point of 0 s represented bacterial culture before the treatment, point 30 s corresponded to the time after the addition of phenolic compounds. The bacterial growth was evaluated after 48 h. The killing kinetics of phenolic compounds was visualized by MS Excel 2016 (Microsoft Corporation, Redmond, WA, USA).

### Seed treatment

The elimination of Xhc was performed on carrot seeds (*Daucus sativa* L.) using seed lots with a high Xhc contamination (approximately 10^6^ CFU g^-1^ of seeds, cv. Sylva), medium Xhc contamination (10^4^ CFU g^-1^ of seeds, cv. Karotina) and a low Xhc presence (10^2^ CFU g^-1^ of seeds, cv. Olympus). The seed treatment was performed in three biological repetitions, using 20 g of seeds for the individual variants.

Seeds were placed into 250 mL conical flasks covered by aluminum foil and treated with 10 mL of 0.0195% carvacrol or 0.0195% thymol solutions (representing the minimum concentrations effective for the elimination of all tested strains *in vitro*) and four and sixteen folds of these concentrations. Non-treated seeds served as a positive control, seeds incubated in pure PBS served as a technical control. The seed treatment was carried out on an orbital shaker (150 rpm) for 30 min. As a comparison, the hot water treatment (52°C for 25 min [14]) and the sodium hypochlorite treatment (NaClO, 1%, 30 min) were included. After each treatment, seeds were air dried for 30 min in a laminar flow box on sterile filter papers.

### Effectivity of treatment on carrot seeds and germinated plants

The surface infestation of carrot seeds was evaluated according to ISTA standard [11]. Briefly, for each variant, the number of 10,000 seeds in three biological repetitions were suspended in 100 mL of sterile pre-chilled seed extraction buffer in conical flasks and soaked overnight in 4°C. Subsequently, the samples were placed on the orbital shaker (200 rpm, 5 min) at room temperature. The obtained seed wash was diluted in ten-fold dilution series and cultivated in triplicates on the semi-selective mTBM medium in the amount of 100 μL. Plates were incubated in a dark at 28°C and 80% humidity in the incubator (Memmert, Buchenbach, Germany) for four days. Six colonies from each treatment and repetition morphologically corresponded to Xhc character were subcultured into sectored plates of YDC (yeast dextrose chalk agar) and incubated for 24 h at 28°C. Obtained bacterial colonies were diluted by PBS (final OD_600_ approximately 0.05–0.08), incubated in 100°C for 10 min and used as a template for endpoint PCR [12]. The positivity of samples with the amplicon corresponding to 355 bp was subsequently tested by TaqMan PCR using the Xhc specific primers and probes from Barnhoorn (MVSXhc3 set), Temple (Xhc-q2 set) and Wu (Wu set) as recommended by ISTA standard.

The efficacy of the treatment against the development of infection in young plants and a possible phytotoxicity of treatments were observed on germinated plants on filter papers. For the estimation of an internal infection, 500 seeds in three repetitions were sown on sterile filter papers and cultivated in plastic boxes for 14 days. After germination, the seed germination percentage was counted. The effect of the treatment on plant vigor was evaluated on 50 plants randomly selected from each repetition through the measurement of plant root and stem sizes. For pathogen detection, 100 plants without testa from each repetition were used. The detection of Xhc in plant samples was performed using the real-time PCR targeting Xhc02 marker [14]. For the quantification of Xhc in positive samples, the real-time PCR assay targeting the *hpaP* gene [28] was used as described above.

### Statistical analysis

The variance analysis and multiple comparisons were used to analyze obtained data. To identify the differences in the reaction of carrots to the seed treatments, the significant level α = 0.05 was used. All statistical analyzes were carried out using the statistical software package STATISTICA (version 12, StatSoft Inc., Tulsa, OK, United States).

## Results

### *Xanthomonas hortorum* pv. *carotae* transmission to germinated plants and real-time PCR detection

The real-time PCR system targeting the *hpaP* gene of the bacterium Xhc positively detected all six Xhc strains with a melting temperature (T_m_) of products from 89.6°C to 90.5°C. Strains of *X*. *arboricola* gained T_m_ of 91.3°C and 92.2°C. The assay was optimized for the detection of Xhc in carrot tissues in concentration of 10^6^–10^1^ CFU mL^-1^ with the efficiency of 105%, values R^2^ = 0.99753 and slope − 3.21 and the cut-off Ct 32 (S2 Table).

The transmission of Xhc to the germinated plants was evaluated on a number of 2000 seeds. From ten repetitions, five subsamples were positive for the Xhc presence, three subsamples were positive based on the Tm of amplified products but regarding the real-time PCR detection limit (Ct 32), they were marked as suspected to be positive (Table 1). Two subsamples were evaluated as negative. The concentration of Xhc in tested subsamples ranged from 2.33 × 10^4^ to 1.36 × 10^1^ CFU mL^-1^. Thus, the seed lot was evaluated as naturally infected by Xhc.

**Table 1:** Real-time PCR estimation of *Xanthomonas hortorum* pv. *carotae* in plants germinated from naturally infected seed lot (cv. Galaxy). Mean values ± SD are presented.

subsample	Mean concentration	Mean Ct	Mean T_m_	Evaluation
Subsample 1	7.32 × 10^1^	29.12 ± 0.50	90.00 ± 0.20	positive
Subsample 2	8.82 × 10^0^	32.07 ± 0.11	90.10 ± 0.10	suspected to be positive
Subsample 3	2.11 × 10^4^	21.22 ± 0.09	90.10 ± 0.10	positive
Subsample 4	3.99 × 10^0^	33.17 ± 0.53	89.73 ± 0.15	suspected to be positive
Subsample 5	-	34.48 ± 1.70	89.50 ± 0.00	negative
Subsample 6	2.33 × 10^4^	21.08 ± 0.05	90.03 ± 0.06	positive
Subsample 7	4.14 × 10^0^	33.12 ± 0.20	89.60 ± 0.00	suspected to be positive
Subsample 8	1.36 × 10^1^	31.46 ± 0.86	89.63 ± 0.15	positive
Subsample 9	1.02 × 10^3^	25.44 ± 0.29	89.83 ± 0.12	positive
Subsample 10	-	No Ct	-	negative

### *In vitro* activity of phenolic compounds

The evaluation of the antibacterial activity of selected phenolic compounds showed the best results for the carvacrol and thymol solutions. For both compounds, the inhibitory effect was observed for the concentration of 0.0098% in the case of Xhc strains NCPPB 230, NCPPB 425, NCPPB 3440, NCPPB 3651 and NCPPB 4410 (Table 2). The Czech strain of Xhc MEND-B-00280 and *X*. *arboricola* strains were inhibited by concentrations of 0.0196%. Interestingly, the minimum inhibitory concentrations were equal to the minimum bactericidal concentrations except for the strain NCPPB 4410, whose MBC value was two times higher than the MIC value. Different results were obtained from the eugenol solution where half of tested strains were inhibited by the 0.0196% solution and the other half by the 0.039% solution. The bactericidal effect was observed mostly in the concentration of 0.078%. Based on the MBC values, subsequent experiments were performed only for the carvacrol and thymol solutions.

**Table 2:** Minimum inhibitory concentrations (MIC) and minimum bactericidal concentrations (MBC) of used strains obtained for carvacrol, eugenol and thymol solutions.

Strain	Carvacrol [%]		Eugenol [%]		Thymol [%]	
	MIC	MBC	MIC	MBC	MIC	MBC
**NCPPB 230**	0.0098	0.0098	0.0196	0.0780	0.0098	0.0098
**NCPPB 425**	0.0098	0.0098	0.0390	0.0780	0.0098	0.0098
**NCPPB 3440**	0.0098	0.0098	0.0390	0.0780	0.0098	0.0098
**NCPPB 3651**	0.0098	0.0098	0.0196	0.0780	0.0098	0.0098
**NCPPB 4410**	0.0098	0.0196	0.0196	0.0196	0.0098	0.0196
**MEND-B-00280**	0.0196	0.0196	0.0196	0.0196	0.0196	0.0196
**MEND-B-00297**	0.0196	0.0196	0.0390	0.0780	0.0196	0.0196
**MEND-B-00300**	0.0196	0.0196	0.0390	0.0780	0.0196	0.0196

**Fig 1:**
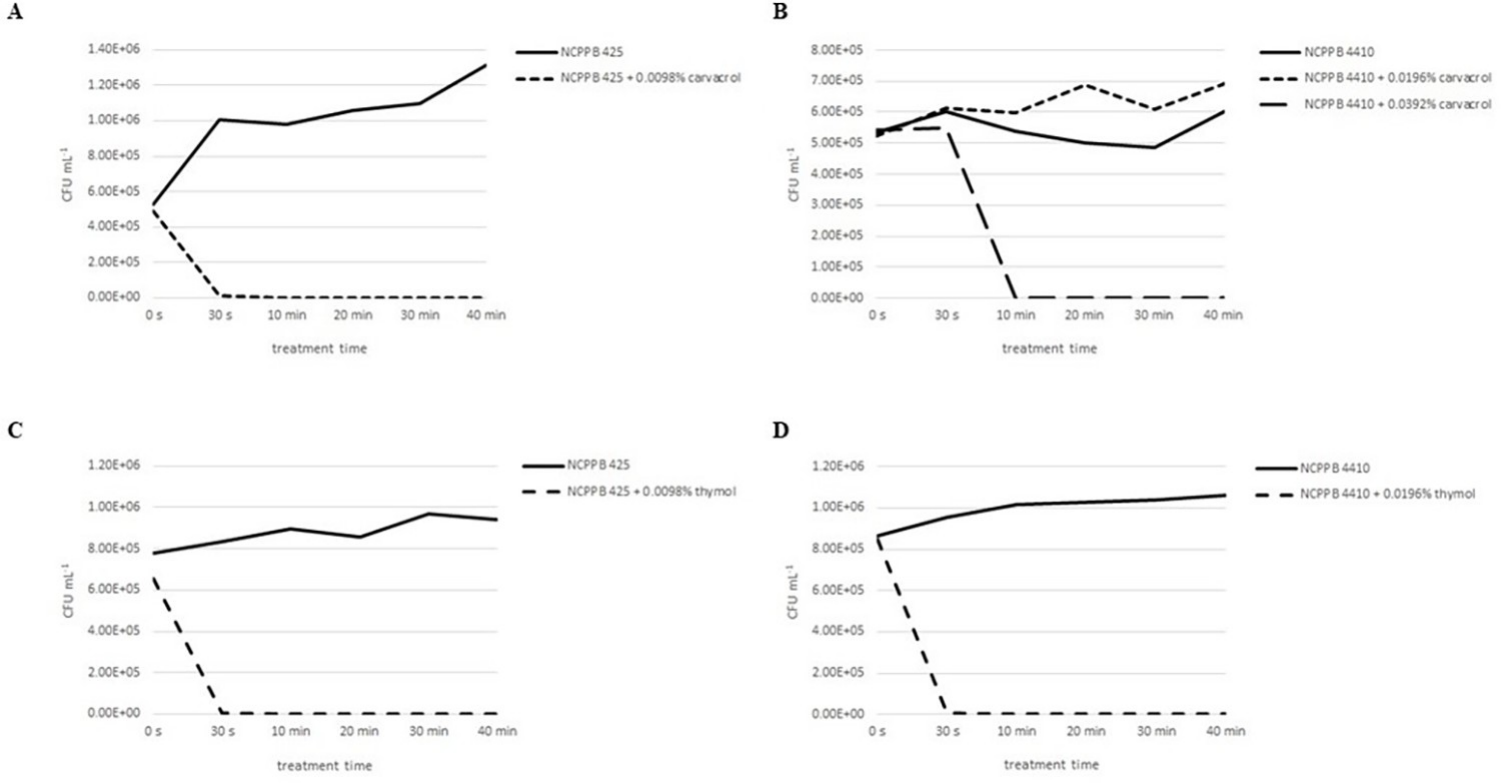
Killing kinetics of carvacrol and thymol to strains NCPPB 425 and NCPPB 4410 [CFU mL^−1^]. Number of CFU per mL counted 48 h after the treatment for (A) strain NCPPB 425 treated with 0.0098% carvacrol, (B) strain NCPPB 4410 treated with 0.0196% and 0.0392% carvacrol, (C) strain NCPPB 425 treated with 0.0098% thymol and (D) strain NCPPB 4410 treated with 0.0196% thymol. Mean values are presented.

Regarding the establishment of an effective time for the phenolic treatment of seeds, the time-kill assay was performed using Xhc strains NCPPB 425 and NCPPB 4410. The cultures treated with carvacrol solution in corresponding MBC values differ in the time necessary for the total elimination of bacterial cells (Fig 1A and 1B). In the case of Xhc strain NCPPB 425, the addition of 0.0098% carvacrol solution decreased the number of Xhc cells from 4.90 ± 0.40 × 10^5^ CFU mL^-1^ to 9.80 ± 0.13 × 10^3^ CFU mL^-1^ and 10 min of the treatment killed all living cells (Fig 1A).

Contrary to NCPPB 425, the treatment of the strain NCPPB 4410 by 0.0196% carvacrol solution (MBC value) did not lead to the death of Xhc cells even after 40 min of treatment (Fig 1B). This result suggests the killing point is closer to 24 h as the MBC value corresponded to the 0.0196% solution. Nevertheless, the treatment with 0.0392% carvacrol (2 MBC value) killed all Xhc cells up to 10 min. The treatment with thymol solution showed a similar reaction of both tested strains when the death of all bacterial cells was achieved up to 10 min after the treatment by corresponding MBC value (Fig 1C and 1D).

Based on the killing kinetics results and predicted effectiveness of the treatment given by the morphological character of carrot seeds, the seed treatment time of 30 min was used for both compounds.

### Effectivity of treatments on carrot seeds and germinated plants

Seed washes diluted as recommended by the ISTA Standard (up to 10^2^) led to the multiple colony presence on the plates, even the semi-selective mTBM medium was used. Many colonies showed morphological character corresponding to the description of Xhc by the producer, meaning yellow, white or white-yellow, glistening, smooth colonies with casein hydrolysis zone. To cover possible variability of natural Xhc strains, type colonies were sampled and cultured on YDC medium. However, the results of endpoint PCR did not show a single amplicon (355 bp) for any of the obtained samples (S1–S3 Figs). A multiple amplicon profile was mostly visualized. The possibility of sample contamination by morphologically similar bacteria was tested by two assays of TaqMan real-time PCR according to the ISTA Standard. Nevertheless, the results of these assays also did not confirm Xhc presence in sampled colonies.

Contrary to these results, the detection of Xhc in germinated plants proved the presence of Xhc in all tested samples in the case of cultivars Karotina and Sylva (Table 3). For cultivar Olympus (low Xhc contaminated seed lot), the elimination of Xhc was achieved by the treatment of 1% sodium hypochlorite, 0.0196% carvacrol, 0.078% carvacrol and 0.313% carvacrol solutions. The remaining treatments did not reduce the presence of Xhc in germinated plants compared to the non-treated and technical control.

**Table 3:** *Xanthomonas hortorum* pv. *carotae* quantification in germinated plants [CFU g^-1^ leaf tissue]. Mean values ± SD are presented.

Sample	Olympus	Karotina	Sylva
Non-treated	1.71 ± 2.14 × 10^4^	1.13 ± 0.66 × 10^4^	1.20 ± 0.44 × 10^4^
PBS	4.16 ± 3.53 × 10^4^	1.29 ± 0.58 × 10^3^	2.24 ± 0.77 × 10^3^
HWT	2.46 ± 0.00 × 10^4^	1.77 ± 0.94 × 10^2^ ^a^	6.20 ± 8.24 × 10^3^
1% NaClO	0 ^a^	2.84 ± 1.23 × 10^2^ ^a^	2.64 ± 2.79 × 10^3^
0.0196% carvacrol	0 ^a^	1.61 ± 0.40 × 10^3^	3.05 ± 1.65 × 10^3^
0.078% carvacrol	0 ^a^	2.94 ± 1.08 × 10^3^	1.20 ± 0.44 × 10^4^
0.313% carvacrol	0 ^a^	3.58 ± 1.16 × 10^3^	1.29 ± 0.37 × 10^4^
0.0196% thymol	1.54 ± 1.27 × 10^4^	4.37 ± 0.71 × 10^3^	9.45 ± 4.09 × 10^3^
0.078% thymol	3.55 ± 1.01 × 10^4^	8.46 ± 4.79 × 10^3^	6.69 ± 2.80 × 10^3^
0.313% thymol	1.71 ± 1.14 × 10^4^	2.99 ± 0.23 × 10^3^	5.73 ± 0.29 × 10^3^

^a^ represents values significantly different from non-treated control (α = 0.05).

In the case of the cultivar Karotina, the use of hot water treatment and 1% sodium hypochlorite significantly decreased the presence of Xhc in carrot tissues compared to the non-treated and technical control but did not eliminate the infection. The quantification of Xhc per one gram of leaf tissue corresponded to the estimated concentration of the bacterium in one gram of seeds. A different situation was obtained for the Xhc highly contaminated cultivar Sylva, wherein the estimated quantity of Xhc (10^6^ CFU g^-1^ of seeds) resulted in an infection of germinated plants at approximately 10^4^ CFU g^-1^ of leaf tissue. The application of seed treatments did not lead to a significant reduction in the quantity of Xhc in carrot tissues. The best results were obtained for the treatment involving 1% sodium hypochlorite and 0.0196% carvacrol solution, demonstrating reductions in infection with values of 2.64 ± 2.79 × 10^3^ and 3.05 ± 1.65 × 10^3^ CFU g^-1^ of leaf tissues, respectively.

**Fig 2:**
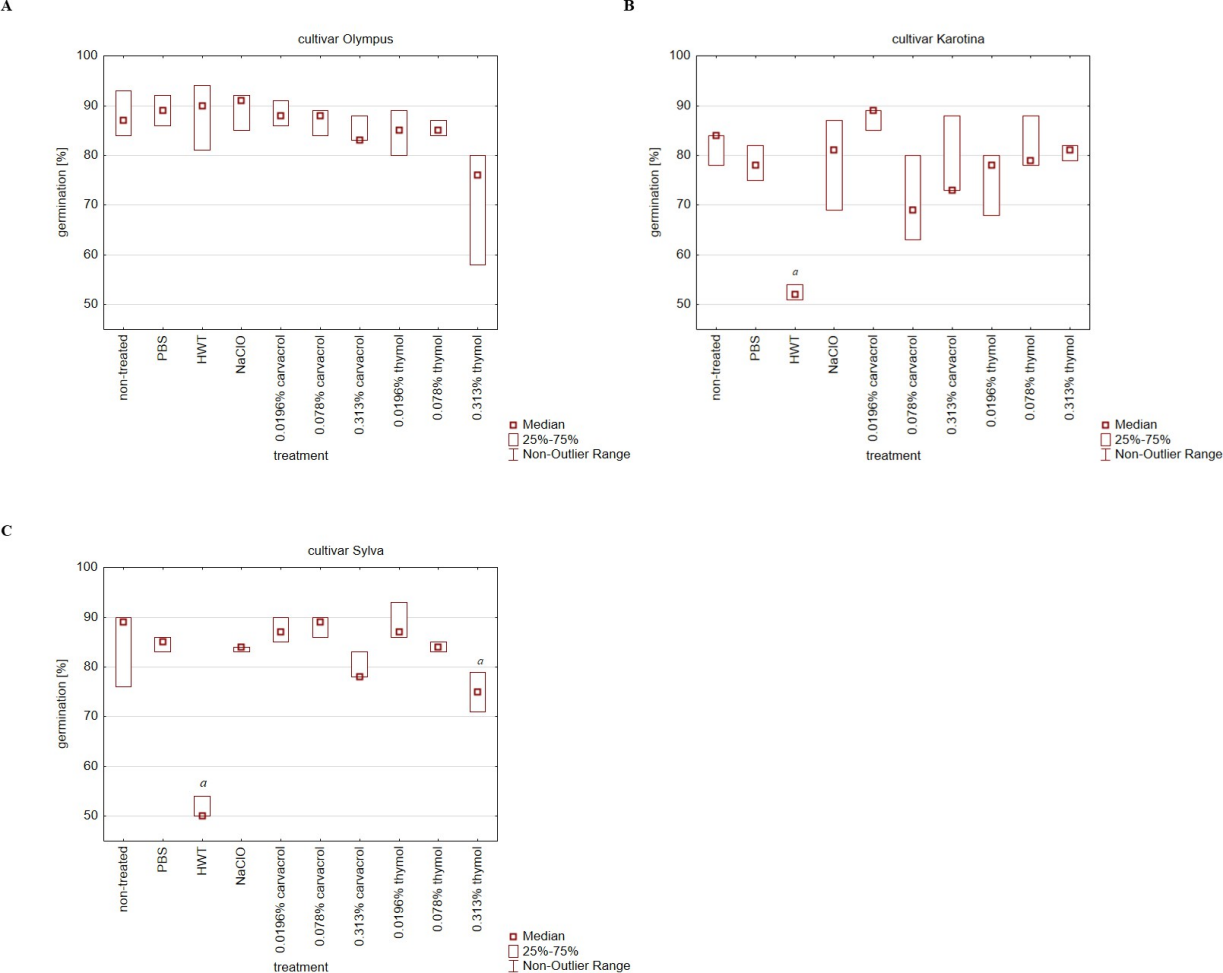
Germination of seeds (%) of three carrot cultivars after individual treatments. (A) cv. Olympus, (B) cv. Karotina and (C) cv. Sylva. Median values are presented. ^a^ represents values significantly different from non-treated control (α = 0.05).

The germination of carrot seeds was not significantly affected by the soaking of seeds in PBS (technical control). For cultivar Olympus, no significant differences in the germination percentage were observed for any treatment (Fig 2A). However, a slightly higher seed germination was observed for seeds soaked in PBS (89.00 ± 2.00%), hot water (88.33 ± 4.89%), NaClO (89.33 ± 2.89%) and 0.0196% carvacrol solution (88.33 ± 1.78%).

In the case of the cultivar Karotina, representing a medium Xhc contaminated seed lot, significantly lower germination was observed for hot water treatment (52.33 ± 1.11%, Fig 2B). Compared to the untreated control with the germination percentage of 82.00 ± 2.67%, the best germination was observed for the seeds soaked in 0.0196% carvacrol solution (87.67 ± 1.78%). The increasing concentration of phenolic compounds had no effect on a gradual decrease or increase in Karotina seed germination.

Different results were obtained for the cultivar Sylva, where an increasing concentration of thymol showed decreased germination of treated seeds (Fig 2C). The 0.313% thymol (sixteen-fold MBC value) led to a significant decrease in seed germination (75.00 ± 2.67%) compared to the untreated control (85.00 ± 6.00%). However, the biggest effect was observed for hot water treatment, where the germination of seeds was reduced to 51.33 ± 1.78%.

The possible phytotoxicity was evaluated also by the measurement of root and stem sizes of germinated plants. Generally, visual deformations, retardation or other morphological changes were not observed for any of the used treatments. However, some plants treated with the highest concentration of thymol solution showed necrosis of leaf tips. The use of PBS and 1% sodium hypochlorite did not have a significant effect on the root and stem sizes of all three tested cultivars. Compared to the non-treated controls, the highest effect of the seed treatment was observed for cultivar Olympus (Table 4). The use of hot water treatment led to significantly longer roots (54.56 ± 10.79 mm) and stems (10.09 ± 2.23 mm) of germinated plants than was measured for the non-treated control (46.72 ± 8.71 mm of root, 7.42 ± 1.52 mm of stem). In contrast, the use of 0.078% and 0.313% carvacrol and 0.0196% thymol caused a reduction of plant root size (37.29 ± 13.48 mm, 30.05 ± 6.90 mm and 30.85 ± 7.79 mm, respectively). Regarding plant stems, the significantly longer stems were obtained after all phenolic treatments, with the longest stems from seeds treated with 0.078% carvacrol (27.63 ± 14.14 mm).

**Table 4:** Root and stem sizes of germinated plants of cvs. Olympus, Karotina and Sylva. Mean values ± SD are presented.

	Olympus		Karotina		Sylva	
	root [mm]	stem [mm]	root [mm]	stem [mm]	root [mm]	stem [mm]
non-treated	46.72 ± 8.71	7.42 ± 1.52	43.16 ± 9.10	10.57 ± 2.49	39.55 ± 9.93	9.50 ± 1.63
PBS	51.42 ± 11.86	8.07 ± 1.46	43.31 ± 11.33	12.22 ± 3.51	43.03 ± 10.46	8.97 ± 1.79
HWT	54.56 ± 10.79^a^	10.09 ± 2.23^a^	30.33 ± 7.94^a^	8.51 ± 2.35^a^	33.71 ± 10.56^a^	10.27 ± 2.53
NaClO 1%	48.53 ± 12.85	7.91 ± 1.56	42.91 ± 12.28	10.39 ± 2.27	40.48 ± 13.21	9.72 ± 2.01
carvacrol 0.0196%	47.15 ± 13.64	10.54 ± 2.47^a^	40.32 ± 6.07	9.46 ± 1.69	39.81 ± 8.58	9.48 ± 1.86
carvacrol 0.078%	37.29 ± 13.48^a^	27.63 ± 14.14^a^	28.82 ± 6.97^a^	9.59 ± 2.17	38.68 ± 8.40	9.23 ± 1.69
carvacrol 0.313%	30.05 ± 6.90^a^	9.19 ± 1.48^a^	27.48 ± 6.64^a^	10.69 ± 1.89	26.53 ± 5.92^a^	11.85 ± 1.51^a^
thymol 0.0196%	30.85 ± 7.79^a^	10.40 ± 1.93^a^	26.41 ± 5.55^a^	10.58 ± 1.39	27.37 ± 5.51^a^	10.65 ± 1.01^a^
thymol 0.078%	50.53 ± 10.30	8.69 ± 1.57^a^	40.77 ± 7.51	9.77 ± 1.89	48.82 ± 12.08^a^	13.47 ± 2.82^a^
thymol 0.313%	48.61 ± 14.48	10.59 ± 1.94^a^	32.05 ± 8.15^a^	16.47 ± 3.94^a^	26.01 ± 10.78^a^	17.17 ± 4.53^a^

^a^ represents values significantly different from non-treated control (α = 0.05).

Similarly to the cultivar Olympus, 0.078% carvacrol, 0.313% carvacrol, 0.0196% thymol, 0.313% thymol treatment and also hot water treatment led to the significantly shorter roots of germinated plants of the cultivar Karotina. The effect on stem size was observed only for hot water treatment (significant reduction to 8.51 ± 2.35 mm) and 0.313% thymol treatment (significant elongation to 16.47 ± 3.94 mm) compared to the non-treated control (10.57 ± 2.49 mm). Plants of cultivar Sylva showed significant elongation of stems and reduction of root size after treatment with 0.313% carvacrol and all used concentrations of thymol except 0.078% thymol that caused root elongation (48.82 ± 12.08 mm). The root reduction was also observed for hot water treatment (33.71 ± 10.56 mm compared to the 39.55 ± 9.93 mm of non-treated control).

## Discussion

The study presents the importance and advantages of the detection of *Xanthomonas hortorum* pv. *carotae* in germinated plants compared to the culturing of seed wash on semi-selective mTBM medium recommended by the ISTA Standard and discusses the potential of carvacrol compound as an alternative treatment to hot water and sodium hypochlorite treatments.

In the first part of the study, the transmission of Xhc from infested seeds into germinated plants was evaluated. For this purpose, instead of the real-time PCR recommended by ISTA for seed testing [11], the assay by Peňázová et al. [28] designed for both pure bacterial cultures and Apiaceae plants was tested. The detection limit of 10^1^ CFU mL^-1^ Xhc in plant tissues proved the same level of sensitivity as the assay for pure bacteria designed by Temple et al. [14]. From ten subsamples, the Xhc transmission was confirmed for five subsamples, with the highest Xhc titre of 2.33 × 10^4^ CFU mL^-1^. Three subsamples were on the border of the detection limit but supposed to be positive based on T_m_. The relatively low incidence of Xhc in plants was most probably caused by low Xhc contamination of used carrot seed lot. Also, the absence of symptoms on germinated plants suggested the initial contamination of seed lot lower than 10^4^ CFU per gram of seeds [3]. Nevertheless, the development of infection in germinated plants highlights the importance of seed sanitations even in the case of low infected seed lots and missing symptoms on germinated plants.

The second part of the study aimed to evaluate the applicability and conditions for use of phenolic compounds treatment in *Xanthomonas hortorum* pv. *carotae* disease management. For the elimination of Xhc, three phenolic compounds were tested. The *in vitro* results of MBC showed higher effect of carvacrol and thymol on the elimination of Xhc growth (range 0.0098% - 0.0196% solutions) than eugenol (0.0196% - 0.078%). The mechanism of disruption of membranes described for carvacrol and thymol seems to be beneficial for the elimination of the *Xanthomonas* genus, contrary to the confirmed non-specific permeabilization of the cytoplasmic membrane and interaction with proteins by eugenol [23]. Similar character of results was reported for *X*. *campestris* pv. *campestris* treated with carvacrol and thymol [21, 22] and also with eugenol [19].

For the estimation of an appropriate seed treatment time, the time killing kinetics of carvacrol and thymol solutions were calculated using two strains of Xhc. Both isolates showed differences in the reaction to the MBC values of carvacrol in time. For Xhc strain NCPPB 425 treated with 0.098% carvacrol, the complete reduction of bacterial growth was obtained after 10 min of treatment. Different reaction was obtained for the less susceptible strain NCPPB 4410, where the MBC value of carvacrol did not eliminate bacterial growth after 40 min of treatment. Moreover, a slight increase in bacterial concentration was recorded after the exposition to 0.0196% carvacrol compared to the growth of non-treated control. This might be caused by the insufficient time required for the growth elimination as the MBC value is estimated after 24 h of the exposition of bacterial culture to the antibacterial agent. Nevertheless, the complete growth elimination was observed for 2 MBC value up to 10 min of the treatment. Contrary to carvacrol, thymol MBCs killed Xhc cells of both isolates 10 min after the exposition of bacterial cells to treatment. The mode of action of these two compounds is similar regarding the interaction with cell membrane [23] so the difference in the killing kinetics was most probably caused by the interaction with different intracellular targets. Obtained results point to the importance of a time-kill assay for the estimation of effective treatment time for MBC values obtained *in vitro* before their application *in vivo*.

The impact of carvacrol’s *in vitro* minimal bactericidal concentration on the eradication of seed infection could not be assessed using seed wash plating. From the obtained seed wash, any of the six type colonies were not positively detected as Xhc. The decrease in the number of obtained Xhc colonies was predicted in accordance with the selection of diverse colony types, as outlined in the comprehensive description of potential Xhc characteristics on mTBM medium provided by the producer. For the cultivar Olympus, characterized by a low Xhc presence in the utilized seed lot (10^2^ CFU g^-1^ of seeds), other bacterial cultures could potentially overgrow the Xhc bacterium in non-diluted seed washes, even when a semi-selective medium was employed. The 10 and 100-fold dilutions most probably did not contain Xhc cells. However, complete loss of the Xhc colonies in the case of the cultivar Sylva with an estimated infection of 10^6^ CFU g^-1^ of seeds was not expected. This result underscores the significance of laboratory workers’ expertise in assessing bacterial colony morphology and the advantage of the estimation of Xhc infection in germinated plants.

Contrary to the seed wash evaluation, real-time PCR successfully identified the presence of Xhc in germinated plants from non-treated samples and technical controls across all evaluated cultivars. A complete reduction of Xhc presence was achieved only with sodium hypochlorite treatment and all carvacrol treatments applied on low infected cultivar Olympus. Despite promising *in vitro* results, treatment with thymol solutions did not lead to the eradication of Xhc cells from carrot tissues even for low contaminated seed lot. For the Karotina cultivar, a significant reduction in infection in plant tissues was only accomplished through the application of hot water and sodium hypochlorite treatments. Nevertheless, the complete elimination of the infection was not achieved. The use of 1% sodium hypochlorite seems to be effective for the elimination of Xhc presence in the case of low or medium infestation of seed lots. The efficacy of low concentrated sodium hypochlorite for seed surface disinfection was described for rice seeds [31] or for lettuce seeds inoculated by *X*. *campestris* pv. *vitians* [18]. Similarly to our results, a significant reduction in lettuce seed germination was not observed. Contrary to sodium hypochlorite, more than 30% reduction in carrot seed germination was observed after hot water treatment for medium and high contaminated seed lots. The use of hot water treatment is generally described as suitable for Xhc elimination from carrot seeds [4, 14]. However, the results from our study suggest the persistence of living Xhc cells on seeds and the establishment of Xhc population in plants under favorable conditions, coupled with low seed germination. The insufficient efficacy of the hot water treatment is in accordance with the study of Sanna et al. or Pečenka et al. [16, 32], where the hot water treatment was used for the elimination of other pathogenic *Xanthomonas* species from seeds. Similarly to our findings, the significant reduction of seed germination after hot water treatment was reported by Pečenka et al. [16] for cabbage seeds highly infected by *X*. *campestris* pv. *campestris*. Regarding the effect of phenolic compounds on seed germination, a significant reduction was obtained only for 0.313% thymol solution applied on highly infested cultivar Sylva. Germinated plants from seeds treated with 0.313% thymol solution exhibited leaf tip necrosis on several plants. Thus, this concentration was evaluated as potentially phytotoxic for carrots.

The impact of seed treatments was also assessed by measuring the root and stem sizes of the germinated plants. In general, only 1% sodium hypochlorite and 0.0196% carvacrol did not affect the length of roots and stems of germinated plants. For the rest of treatments, the reduction of root lengths and elongation of stems was observed. Nevertheless, the changes in root and stem lengths might also be caused by the change of microbial spectrum on the seed surface. The effect of treatments on other pathogenic or beneficial microorganisms were not evaluated in this study.

## Conclusion

Based on the obtained results, the use of 1% sodium hypochlorite and 0.0196% carvacrol solution are suggested as appropriate for use in the management of Xhc presence in low contaminated seed lots. Contrary to sodium hypochlorite, the volatile character of the carvacrol compound may bring an advantage in practical use, as no additional costs are required for the liquidation of used treated water. On the other hand, the low solubility of carvacrol suggests its use for smaller volumes of seeds to maintain the efficacy of the treatment. The 0.0196% carvacrol treatment might be applied to hybrid lines suspected of Xhc presence with higher efficacy to eliminate infection from germinated plants than hot water treatment and with no additional costs compared to sodium hypochlorite treatment. For seed lots with medium and high Xhc contamination, the use of 1% sodium hypochlorite remains the best option in terms of effectively reducing Xhc infection without impacting the lengths of the roots and stems of germinated plants.

## Supporting information

S1 Fig Endpoint PCR results for Xhc detection in cultures originated in seed wash of cv. Olympus. (TIF)

S2 Fig Endpoint PCR results for Xhc detection in cultures originated in seed wash of cv. Karotina. (TIF)

S3 Fig Endpoint PCR results for Xhc detection in cultures originated in seed wash of cv. Sylva. (TIF)

S1 Table *Xanthomonas* strains used in the study. (XLSX)

S2 Table Real-time PCR results of standards used for the quantification of Xhc in carrot tissues. (XLS)
