# Supplementary figures and images for: Management of bacterial blight of carrots by phenolic compounds treatment
Source: PLoS One. 2024 Apr 1;19(4):e0299105. doi: 10.1371/journal.pone.0299105 (PMC10984397; doi:10.1371/journal.pone.0299105)

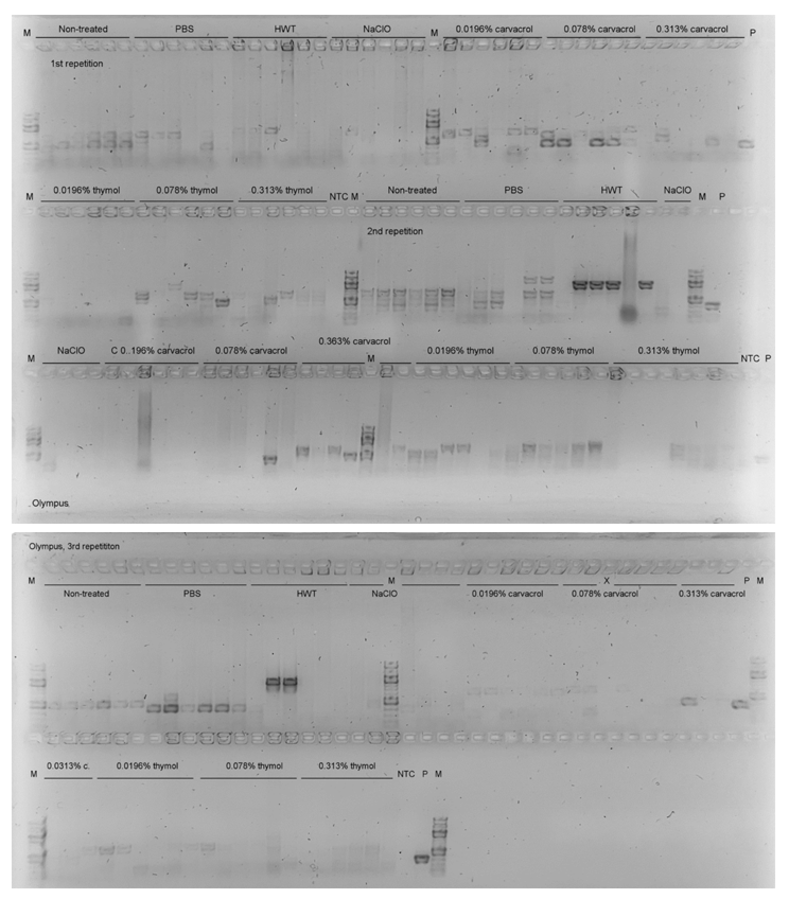

Supplement: S1 Fig — (TIF) [file pone.0299105.s001.tif]

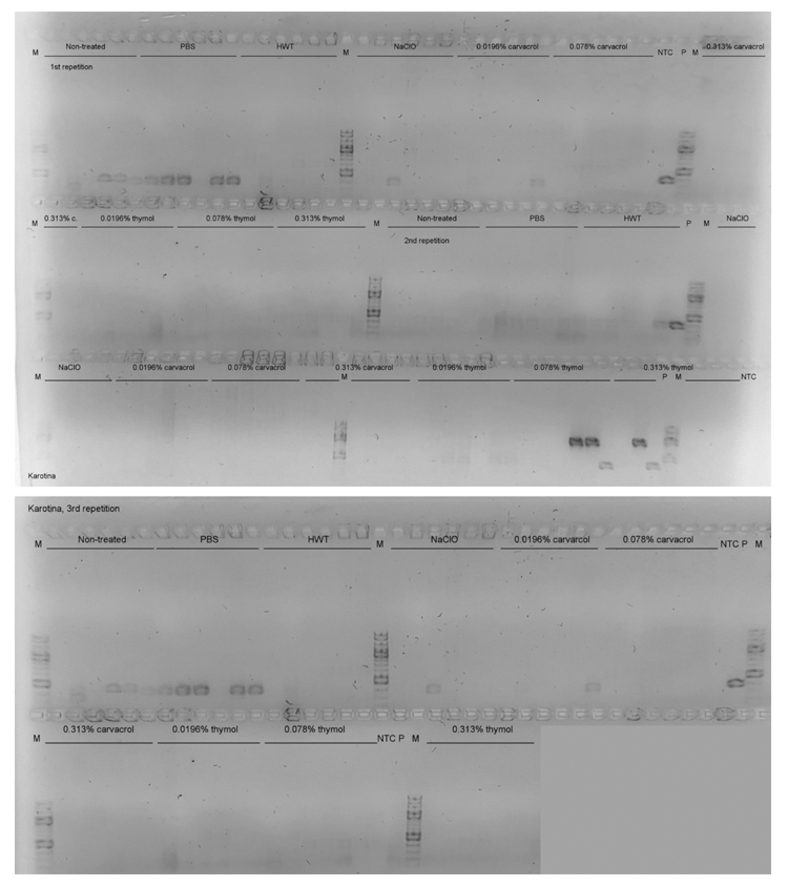

Supplement: S2 Fig — (TIF) [file pone.0299105.s002.tif]

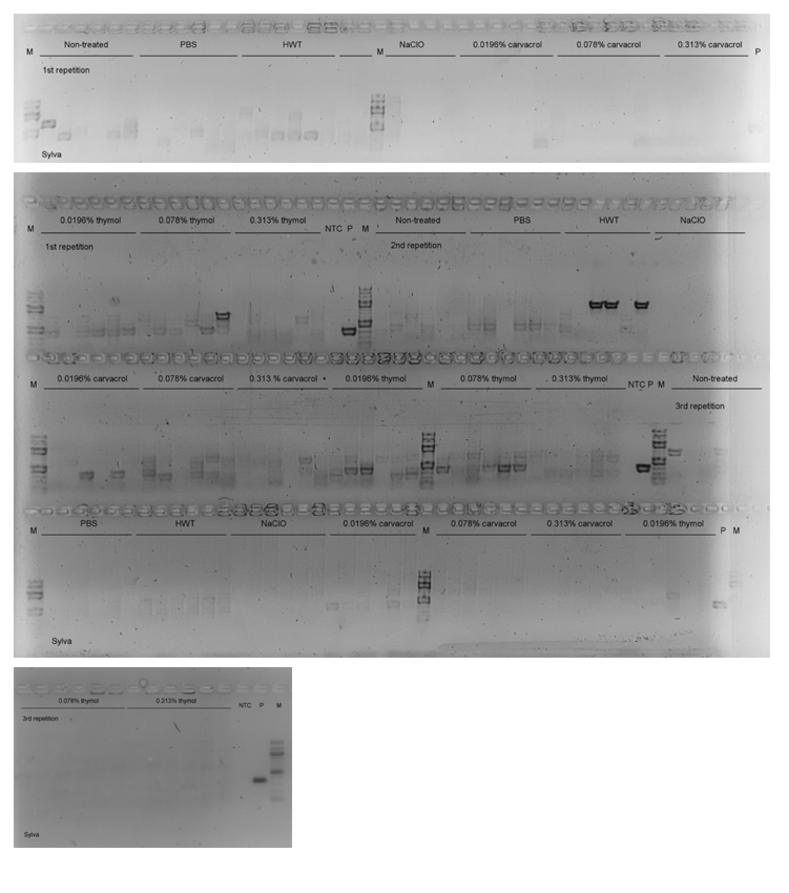

Supplement: S3 Fig — (TIF) [file pone.0299105.s003.tif]
